# Heredity in Nervous Diseases and Its Social Bearings

**Published:** 1914-01

**Authors:** W. A. Gardner

**Affiliations:** Atlanta, Ga.


### HEREDITY IN NERVOUS DISEASES AND ITS SOCIAL BEARINGS.

By W. A. Gardner, M. D., Atlanta, Ga.

Every topic into which heredity enters can now in view of the recent advances in this study, be viewed in a new light. Mental abnornality may be divided into two sorts; lack of development and weakness. The development of an individual is, a complex of processes which work individually through the production of specific organs, and collectively to the development of the individual as a whole.

Modern studies in heredity indicate that, typically each part develops to a certain extent independently of every other part, at least in the early stages. Later the secretions of the thyroid and thymus, testes and ovaries, and doubtless other organs exert a general or a specific influence on development. Finally, the insufficient nutrition of the embryo or the presence of certain poisons may exert a wide-spread inhibiting effect on developmental processes.

Even starvation exerts a somewhat selective influence since all organs are not equally retarded, and there is reason for concluding that malnutrition and poison have a different effect on different persons, retarding those in whom the internal impulse to development is weakest.

Failure of mental development may, on the classification adopted, be regarded as of four sorts; first, intrinsic, and general producing idiots and creations, second, entrinsic and specific producing the feeble minded, third, extrinsic and general resulting (it is alleged) in absortions and miscarriages, fourth, extrinsic and specific causing (perhaps) some cases of harelip, eunuchoidism and perhaps some other defects. Of the four classes, the second is commonest.

From a practical point of view, feeble-mindedness is the failure of developmnt of specific mental traits, the determiners for which are not present, in, and have never been acquired, by the germ-plasm. The defects are a direct inheritance from remote ape-like ancestors in which the traits had not yet been acquired. The feeble-minded have gained most human traits, but not al the mental ones.

The group of the mentally weak include those persons who, under the influence of stress break down mentally. The stress reveals the hidden weakness but does not cause it. Studies of the families of epileptics, dementie praecox and manic-depressives reveal the fact, that they belong to strains with mental weakness. If we inquire whence those strains arose, no final answer can be given, but the suggestion may be made. Every person has a weakest point, we are not like the "One Hoss Shay." There is evidence that there are human strains, that are not resistant to attacks on the respiratory system, others whose weakness is in the circulatory system, others whose alimentary tract is the first to give way and others whose nervous mechanism cannot stand great stress. And those persons who go insane belong for the most part, to this last set. We might say that the mentally weak include those mental adjustments which have not kept pace with the increasing demands of our civilization. tion.

A brief statement here of the Mandelion theory is worth including on account of the clearness and simplicity of statement. The Mendeloin theory assumes that the total inheritance of an individual is divisable into unite characters, each of which is, as a general rule, inherited independently of all other characters and may, therefore, be studied without reference to them. The inheritance of any such character is believed to be dependent upon the presence in the germ-plasm of a unit of substance called a determiner. With reference to any given character, the condition in an individual may be dominant or recessive. The character is dominant when depending upon the presence of its determiner, in the germ-plasm, it is plainly manifest, and if recessive when owing to the lack of its determiner, in the germ-plasm, it is not present in the individual under consideration.

From this point of view, the origin of a feeble-minded child, which seems so like the visitation of an offended God is merely the necessary cropping out of inherited traits, and the origin of a functionally insane person is, within limits determined by a fortuitous combination of weak determiners.

Fere, says: "what favors the multiplication of neuropathic degeneracies is that neurotic persons of any kind have a remarkble tendency to seek one another, and this pathological selection continues to swell the number." Ireland, very truthly remarks, "when one learns the anatomy of the heart he is at no loss to understand how it acts as a pumping machine, but our knowledge of the structure and mechanism of the brain does not throw much light on its functions, knowledge of the arteries, capillaries and veins makes the circulation of the blood clear, knowledge of the anatomy of an organ takes us far into its physiology. Morphology and histology of the brain make us but little wiser as to how its structure is adapted for its completed functions. It is like a scroll which we cannot decipher. We know that it has a meaning and that if the character were altered or the lines transposed, the significance would be different or there would be no significance at all. Mott is of the opinion that a neurotic temperament may be manifested in many different ways, like conduct or behavior, and this neurotic temperament may be the first evidence of any degeneration in the stock. Unsound stock may have successful men in the eyes of the world, but these may readily form the first steps in the process of degeneration for avarice and normal guile, which made them pillars of society, may come out in the next generation as gross ciminality or insanity.

Leaving for a moment the matter of the neuropthic strains, let us consider their relation to society. Society is an organization of a gregarious species for development of common interest through co-operation and division of labor. Social progress implies mutual understanding, ability to co-operate and play one's part well, while regarding the good of others. Society is built on human behavior and the behavior of an individual at any time is his reaction to the stimuli that fall on him The reaction is determined not only by the stimulus but also by the individual make-up both that part which come through heredity, and that which comes through training (which in turn is largely influenced by heredity). Society has decided that certain kinds of behavior—reactions are, on the whole good for the development of society, and that certain others are bad. The inability to learn to count, to read, to write, to distinguish colors, the lack of ambition, of foresight, of industry, of sex control and control over other emotions, such as anger, fear, hatred, suspicion and revenge, the tendency to seclusiveness, to active shyness, to conceit, to periods of depression, to exaltation, to expansive ideas, to loss of memory, to loss of orientation, to cruel treatment to animals and children, all of these reactions are (inheritable) and are bad for society which has labeled them (bad behavior). The persons who behave this way are incompatible with a safe and progressive organization and they should in the worst cases, be segregated to protect society, and above all that they may not reproduce their kind.

Finally, since most if not all kinds of nervous diseases have their hereditary element, is is clearly desirable that such diseases should not be perpetuated by unfit matings, such as by two strains containing nervous weakness. In order that such marriages may not occur, the fact of neural weakness in a family strain should be known to the one comtemplating marriage into that strain. It is a duty of a physician in the exercise of his broad function as eugenist to warn young people who are contemplating a choice that is clearly unfit. Inheritable traits, are not personal property. They come to the individual from down the ages out of the society of the part, they will be disseminated if the person or his fraternity have children, into the society of the future. What right has the momentary possessor of the trait to claim it as his private or personal affair? A knowledge of his traits belongs to society, is it not wrong for the profession to maintain these ideas of the private nature of inheritable traits?

Those who should not marry and should not transmit are those who are possessors of undesirable traits. If when an apparently desirable person happens to transmit to his or her offspring undesirable traits, such offspring should be prevented in turn from begetting or bearing children even though he may produce some healthy pregnancy.

Everything, every movement, has to have a beginning and their decision not to marry people having a communicable disease of immoral origin is a splendid idea as far as it goes, and we physicians should unhesitatingly do everything in our power to aid in this movement, but in time it should be possible to forbid the issuance of a license, to any person having a communicable disease, no matter of what origin. That takes in everything from diseases of personal immoral origin to diseases of inheritance. Then, and only then will we begin to stem the tide of criminal, degenerate feeble-minded, epileptic and insane, which is so purely polluting and threatening extermination of our race.

As to the improving of human strains, our knowledge of the subject as yet is infimitesimal, and as to experience we have none. All we actually know about it is from analogous reasoning, based on the crossing of animals and plants. But we also know that certain race mixtures produce fine types of normal manhood and womanhood and that certain other race mixtures debase these types, and their products are subnormal and inferior to the parent races in stability and equilibrium. But to breed men and women on the scientific and progressive scale that animal and plants are based on, we know but little about it.

Before anything of the kind can be attempted our laws of marriage must undergo beneficial alterations pseudo prudishness be relegated to where it belongs, and the reproduction relations of men and women be placed on the high moral plane which the Creator originally ordained.

It is said that there has arisen in all civilized societies in various ways and for various reasons, a grave and growing danger to the future of the human race. Not only as the nat ural elimination in the inferior stocks not been checked, but most societies permit the elimination of the superior. A sociologist of high character and standing has stated that our public institutions for the insane, the criminal, and the defective are "monuments of our own folly" and that until the source of the trouble is eliminated, we must continue to build jails, penitentiaries, etc. in order to make provision for all who ought to be within their walls. He states that it pays better to provide permanent care for a feeble-minded boy or girl during the entire reproductive period, than to support their offspring admits of no argument, and each years' delay in their permanent segregation means added expense, added difficulties and added misery.

The United States for nearly half a century has been the dumping ground for the criminal, pauper, and feeble-minded emigrants of Southern and Eastern Europe, which in connection with out nation's production of the same, has made us the "melting pot of crime, pauperism and feeble mindedness" of such vast proportions that we are astounded and thunderstruck by simply glancing over it.

But as Matthew Arnold says: "there is abroad in the universe a power not within ourselves that makes for righteousness," so let us pray that it will make for the assured protection, tender care and proper training of the feeble minded in our midst.

Howell Park Sanatarium, Atlanta, Ga.

